# Peroneal artery perforator flap for the treatment of chronic lower extremity wounds

**DOI:** 10.1186/s13018-017-0675-z

**Published:** 2017-11-10

**Authors:** Liang Cheng, Xiaqing Yang, Tingxiang Chen, Zhijie Li

**Affiliations:** Department of Hand and Plastic Surgery, The Second Affiliated Hospital and Yuying Children’s Hospital of Wenzhou Medical University, 109 Xue Yuan Xi Road, Wenzhou, Zhejiang China

**Keywords:** Peroneal artery perforator flap, Chronic lower extremity wounds, Reconstructive

## Abstract

**Background:**

Reconstruction of chronic lower extremity wounds remains challenging. These wounds are mainly associated with diabetes mellitus, infections, and osteomyelitis. Although several reconstructive techniques are available, the peroneal artery perforator flap has unique advantages.

**Methods:**

In this study, we discuss our experiences with peroneal artery perforator flaps in 55 patients who had suffered from chronic lower limb wounds. The size of the defect, comorbidities, etiology, flap size, and complications were recorded and analyzed based on a retrospective chart review.

**Results:**

All 55 flaps survived. In two cases, small superficial necrosis occurred, one of which healed with conservative treatment and the other was reconstructed with split thickness skin grafts. Partial necrosis was observed in nine cases, seven of which were covered with split thickness skin grafts and the remaining two sutured directly after adequate debridement. Vascular compromise was observed in one patient, which was salvaged successfully by performing an exploratory procedure and releasing a few sutures. No complications were seen in the remaining 44 cases.

**Conclusion:**

The peroneal artery perforator flap is a reliable option for reconstruction of chronic lower extremity wounds.

## Background

Reconstruction of chronic lower extremity wounds remains a challenging task, particularly in patients with circulation problems. Various options including local flaps, free flaps, and muscle flaps have been used for reconstruction in these cases; however, rebuilding techniques to enhance outcomes have not been identified [1–6]. Before utilizing local flaps, free flaps, or pedicle flaps, surgeons should reduce the amount of soft tissue and determine the clinical application subjected to their limited reach. In 1984, Yoshimura et al. [7] introduced the peroneal artery perforator flap. The perforator flap is based on the concept that skin can be divided into angiosomes [8]. Indeed, for the perforator approach, the recipient area has a flexible rotation with remarkable applicability, and the flap is nourished by perforator vessels that arise from a deep vascular system [9]. Compared with the traditional flap or workhorse flap (such as the anterolateral thigh flap), the peroneal artery perforator flap decreases bleeding, preserves muscle function, has a multiform flap design, and enhances mobility of the flap [10]. Besides, a peroneal septocutaneous or musculocutaneous perforator stems from the parent vessel, which directly supplies the overlying skin, and the flap helps preserve the peroneal vessel system. Due to these advantages, peroneal artery perforator flaps are a suitable choice for the treatment of chronic lower extremity wounds.

In this report, we describe our experiences with 55 patients suffering from chronic lower extremity wounds who underwent surgical reconstruction with peroneal artery perforator flaps.

## Methods

This study was performed in accordance with the ethical standards of the Declaration of Helsinki. Ethics approval was obtained through the Hospital’s Regional Ethics Committee, and all patients gave informed consent prior to inclusion in the study.

A retrospective analysis was performed using the medical records of 55 patients who underwent lower extremity wound reconstruction using peroneal artery perforator flaps between May 2008 and September 2015. For each patient, the following data were collected and recorded: age, sex, etiology, size of the defect, comorbidities, dimension of the flap, complications, and follow-up. The patients included 43 males and 12 females, and their ages varied from 3 to 78 years, with an average age of 48.87 years. A total of 22 patients suffered from open tibia/fibula/ankle/calcaneus fractures with associated infection; open fracture in 14, machine crash in 1, crash-injury in 6, osteomyelitis in 4, tumble in 1, scald in 1, skin ulcer in 1, chronic tophus gout in 1, foot mass in 1, and 3 cases of an Achilles tendon rupture associated with infection. The soft-tissue defect was located on the calcaneus in 7 cases, the malleolar area in 16 cases, around the knee in 4 cases, the acrotarsium area in 10 cases, and the lower extremity in 18 cases. Defect sizes ranged from 1.5 to 300 cm^2^. The wounds were debrided an average of 2.98 times (range, 1–8 times). All cases were performed with vigorous debridement, after which the peroneal artery perforator flaps were applied.

### Surgical technique

A Doppler probe was used preoperatively to locate the peroneal artery and the most appropriate perforator vessel. Under a combined spinal epidural analgesia, patients were placed in a supine position with the injured legs slightly abducted and the thigh under tourniquet control. After vigorous debridement, the outline of the flap was drawn based on the size and shape of the defect. Flap design and orientations around the sited perforators ensured adequate length and width so that the flap could be harvested. Flap dissection was initiated along the anterior side of the flap down to the crural fascia and was performed in the same fashion on the posterior side. Subfascial dissection was performed laterally until the septocutaneous perforator or musculocutaneous perforator was identified. Because the process of the musculocutaneous perforator is often twisted, dissection is performed punctiliously to avoid perforator injury. After confirming that the perforator was a branch of the peroneal artery, the flap was harvested. The raised flap was able to rotate around the perforator and adapt to the defect. In a small number of cases, split thickness skin graft (STSG) derived from the thigh was required to cover the defect with the peroneal artery perforator flap, and most donor sites were closed. Over-tight bandaging was avoided to limit vascular embarrassment, and a window was made in the dressing to observe the flap. Before ambulation was achieved, anticoagulation treatment with low weight molecular heparin was introduced. Postoperatively, all cases received appropriate antibiotic therapy and symptomatic rehydration support treatment. All patients were coached periodically until the wound site was achieved and the donor had healed. Generally, flap sutures were dismantled on the 14th postoperative day.

### Case 4

**Fig. 1:**
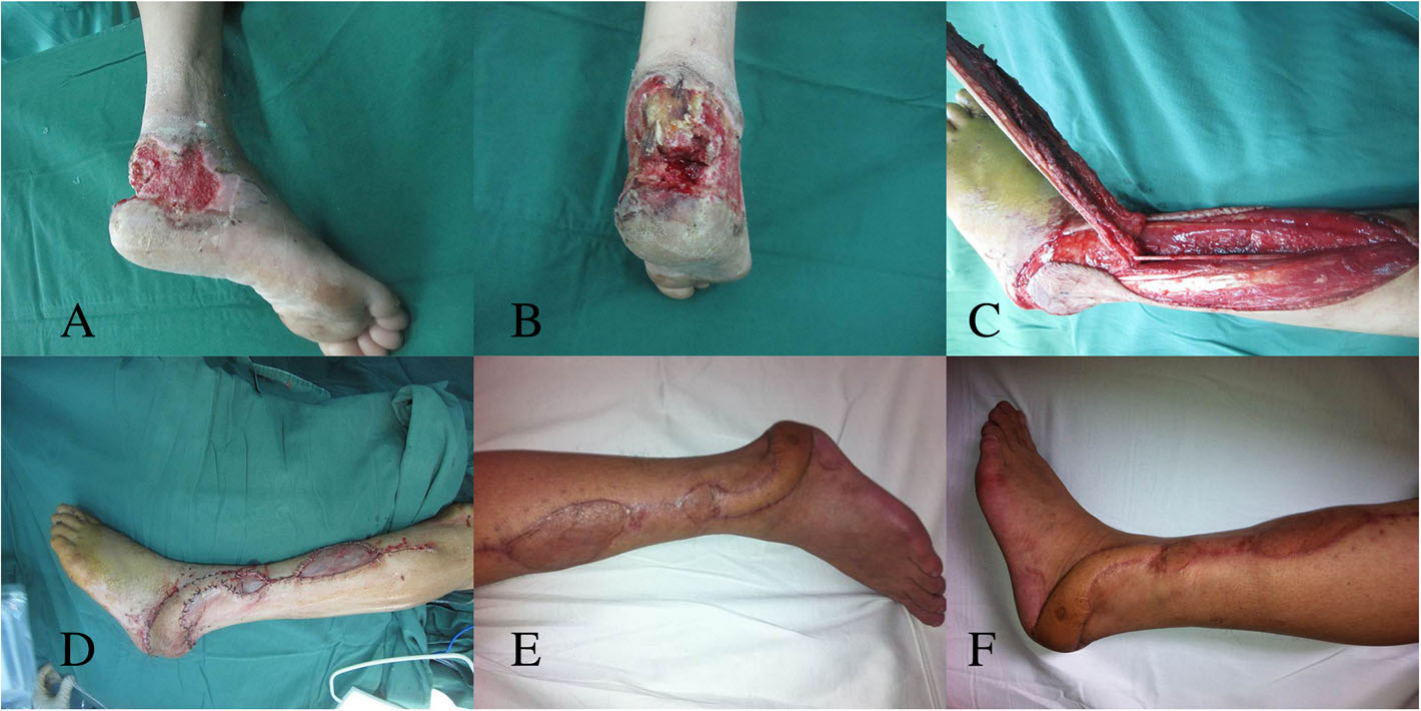
**a**, **b** A defect with exposed calcaneus in the heel. **c** Harvesting the peroneal artery perforator flap. **d** Early postoperative view. **e** Appearance 6 months after operation. **f** Follow-up at 12 months

A 50-year-old female suffered a soft tissue defect around the ankle joint due to a traffic accident. After radical debridement, a peroneal artery perforator flap measuring approximately 20 cm × 7 cm was elevated from the ipsilateral lower leg. The flap was subsequently inset into the defect at 150 degrees based on the pivot of the perforator. The donor site was closed by combining the split thickness skin graft. Postoperative recovery was uneventful; the flap survived completely. A satisfactory result was obtained at 1-year follow-up (Fig. 1).

### Case 15

**Fig. 2:**
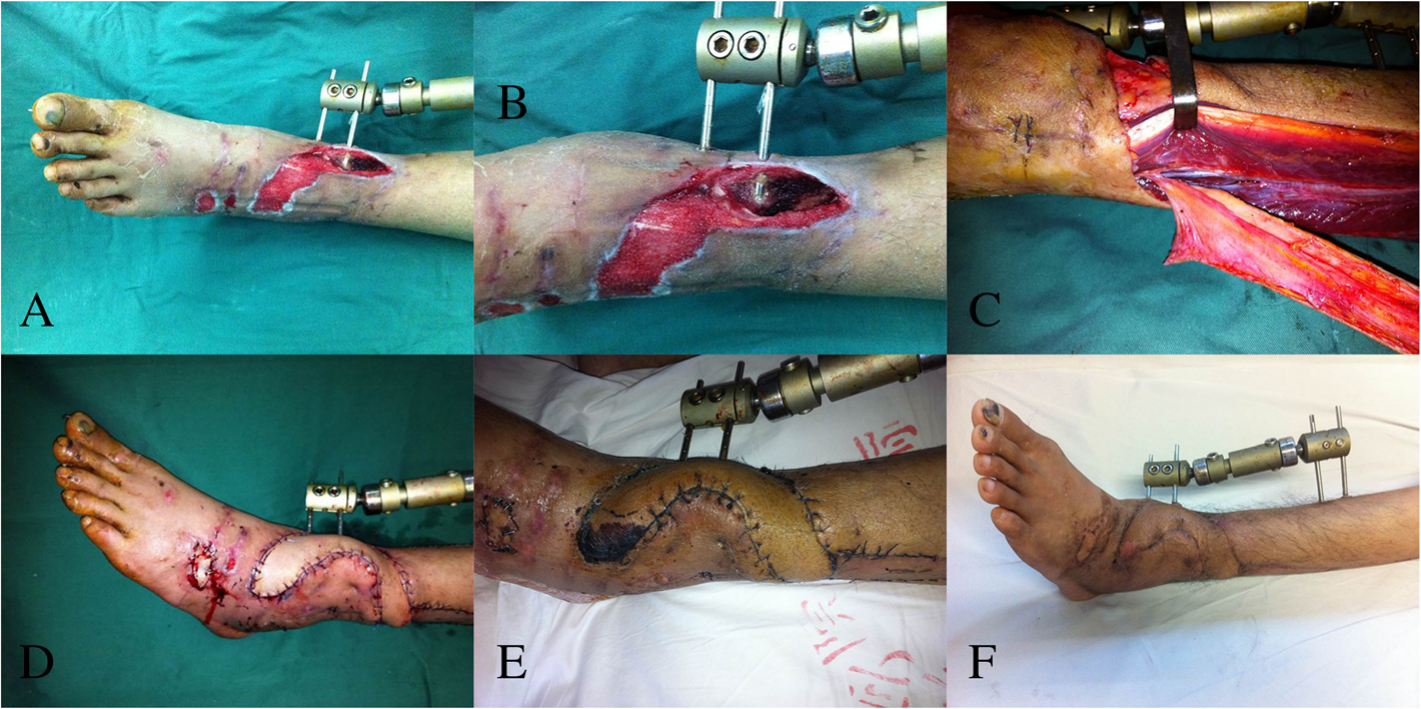
**a**, **b** A defect with exposed fracture in the distal of lower limb. **c** Raising the peroneal artery perforator flap. **d** Early postoperative view. **e** Appearance 10 days after operation. **f** Follow-up at 12 months

A 38-year-old man developed traumatic bone exposure with a soft tissue defect after suffering an open tibia and fibula fracture. After debridement of the necrotic tissue, a peroneal artery perforator flap measuring 15 cm × 3 cm was raised from the same leg and transferred to the defect. The donor site was closed. Postoperatively, the distal part of the flap showed partial necrosis and was treated with dressing changes. Fortunately, the flap survived, and the patient was satisfied with the appearance (Fig. 2).

### Case 20

**Fig. 3:**
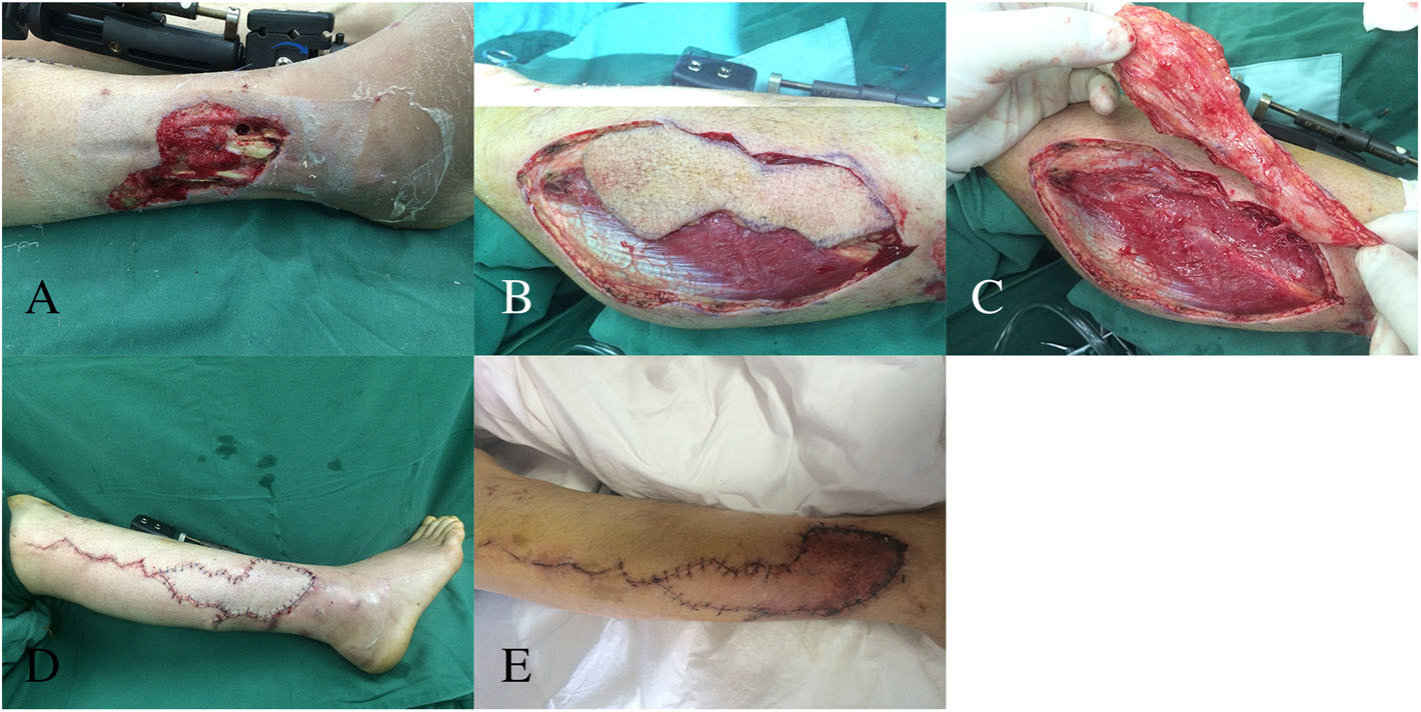
**a** A defect with exposed fracture in the distal of lower limb. **b** Dissected the peronal artery perforator flap. **c** Elevating the peronal artery perforator flap. **d** Early postoperative view. **e** Appearance 7 days after operation

A 46-year-old man had a traumatic soft tissue loss of the lower leg with exposure of the bone. To restore function, a peroneal artery perforator flap measuring 18 cm × 4 cm was harvested and transferred to the defect. The donor site was closed, and the transferred tissues survived completely (Fig. 3).

## Results

From May 1997 to September 2015, 55 peroneal artery perforator flaps were performed in patients with chronic lower extremity wounds. The details of all patients are presented in Table 1. The average operating time was 132 min, and the flaps ranged in size from 1.5 to 260 cm^2^. All 55 flaps survived. In two cases, small superficial necrosis occurred, one of which healed with conservative treatment and the other reconstructed with STSG. Partial necrosis was observed in eight cases, six of which were covered with STSG and the remaining two directly sutured after debridement. Vascular compromise was only observed in one patient and was salvaged by performing an exploratory procedure and releasing a few sutures. In one patient, the ultra-thin flap procedure was performed at 6 months postoperatively due to a bulky appearance. No complications were seen in the remaining 43 cases. The length of hospital stay ranged from 7 to 80 days (average of 33.36 days). Follow-up ranged from 7 to 45 months, with an average of 25.9 months. Ultimately, all patients were satisfied with the functional results and could walk comfortably.

**Table 1:** Data of the patients

Patient No.	Age/gender	Etiology	Size of defect (cm)	Comorbidities	Dimension of flap (cm)	Complication	Secondary procedure	Follow-up (months)
1	76/M	OFSI	6 × 4	HTN	4.5 × 2	None	None	18
2	67/M	ATRSI	4 × 6	Arteriopathy	4 × 6.5	None	None	13
3	7/M	OFSI	4 × 5	None	4 × 5	None	None	10
4	50/F	Open fracture	20 × 4	HTN	20 × 7	None	None	7
5	44/F	Skin ulcer	6 × 6	DM	5 × 5	None	None	20
6	54/M	Open fracture	8 × 6	DM/arteriopathy	8 × 6	Partial necrosis	Debridement, suture	19
7	50/M	OFSI	4 × 3	None	4 × 3	None	None	12
8	47/M	OFSI	14 × 8	None	14 × 8	Bulky appearance	Ultra-thin flap procedure	23
9	69/F	Osteomyelitis	12 × 8	DM	13 × 8	Partial necrosis	STSG	14
10	59/M	Crush-injured	10 × 5.5	None	10 × 6	None	None	28
11	23/M	Open fracture	20 × 10	None	20 × 13	None	None	27
12	51/M	Tumble	10 × 10	None	9 × 8	None	None	33
13	67/F	Foot mass with secondary infection	3 × 5	Arteriopathy	5.5 × 3.5	None	None	31
14	28/M	Crush-injured	3 × 4	None	3 × 4	Vascular compromise	Exploratory procedure, releasing of a few sutures	45
15	38/M	OFSI	6 × 2	None	15 × 3	None	None	19
16	55/M	OFSI	6 × 5	None	6 × 5	None	None	25
17	51/M	Open fracture	11 × 4	None	12 × 6	None	None	9
18	48/M	OFSI	8 × 10	None	12 × 9	None	None	22
19	63/M	OFSI	15 × 7	DM	15 × 8	None	None	37
20	36/M	OFSI	15 × 8	None	12 × 6	None	None	18
21	30/F	Open fracture	6 × 7	None	6 × 6	None	None	9
22	46/M	OFSI	8 × 5	None	18 × 4	None	None	41
23	66/M	OFSI	6 × 3	HTN	7 × 4	Partial necrosis	STSG	24
24	40/M	ATRSI	8 × 9	None	8 × 9	None	None	27
25	37/M	ATRSI	3 × 2	None	4 × 3	None	None	22
26	44/M	Machine crashed	6 × 6	HTN	8 × 6	Partial necrosis	STSG	43
27	62/M	OFSI	9 × 4	Arteriopathy	9 × 4	None	None	36
28	44/M	Open fracture	5 × 15	Arteriopathy	5 × 25	Superficial necrosis	Dressing change	30
29	60/M	Chronic tophus gout	3 × 4	Chronic tophus	5 × 6	Partial necrosis	STSG	38
30	58/F	OFSI	1 × 1.5	None	1 × 1.5	None	None	26
31	48/M	OFSI	10 × 8	DM	12 × 10	Superficial necrosis	STSG	16
32	63/M	Crush-injured	8 × 8	None	8 × 9	None	None	38
33	53/F	Open fracture	8 × 5	None	8 × 7	None	None	24
34	36/M	OFSI	5 × 4	None	6 × 5	None	None	30
35	71/M	Osteomyelitis	5 × 4	DM/HTN	5 × 4	None	None	22
36	63/F	Open fracture	8 × 4	None	9 × 4.5	None	None	28
37	78/M	Open fracture	15 × 15	DM/HTN/arteriopathy	15 × 10	Partial necrosis	STSG	42
38	47/M	Open fracture	20 × 15	None	20 × 10	None	None	36
39	3/F	Open fracture	12 × 7	None	12 × 7	None	None	24
40	56/M	OFSI	6 × 4	None	6 × 4	None	None	22
41	60/M	OFSI	5 × 2	None	5 × 3	None	None	33
42	20/M	Open fracture	12 × 8	None	16 × 8.5	None	None	27
43	72/M	OFSI	10 × 5	DM/HTN	12 × 6	None	None	37
44	60/M	OFSI	3 × 11	None	3 × 11	None	None	27
45	61/M	OFSI	4 × 6	DM/arteriopathy	5 × 7	Partial necrosis	Debridement, suture	18
46	60/F	OFSI	4 × 4	None	4 × 4	None	None	22
47	47/M	Open fracture	12 × 10	None	12 × 10	None	None	23
48	44/F	OFSI	12 × 10	None	16 × 10	None	None	47
49	53/M	Osteomyelitis	7 × 5	None	7 × 5	None	None	28
50	49/M	Osteomyelitis	4 × 5	None	5 × 4	None	None	34
51	5/F	Scald	5 × 6	None	5 × 7	None	None	19
52	44/M	Crush-injured	5 × 7	None	5 × 7	None	None	31
53	28/M	Crush-injured	15 × 10	None	15 × 10	None	None	27
54	39/M	Open fracture	15 × 7	None	15 × 8	None	None	16
55	58/M	Crush-injured	15 × 10	DM/HTN	15 × 10	Partial necrosis	STSG	7

Notes: *M* male, *F* female, *OFSI* open fractures with secondary infection, *ATRSI* Achilles tendon rupture with secondary infection, *DM* diabetes mellitus, *HTN* hypertension, *STSG* splint thickness skin graft

## Discussion

Reconstruction of soft tissue defects overlying the lower limbs remains a significant challenge, as this region is typically associated with exposure of tendon or bone and metal fixation of fractures. Wound healing is markedly prolonged (leading to chronic wounds) due to a lack of adequate soft tissue coverage and a decrease in distal perfusion of the lower limbs. Since the freestyle perforator construct and perforasome theory were proposed, the use of local flaps has increased [11]. In addition, because adjacent tissue is typically involved and massive edema formation prevents adequate mobilization, access to a local flap is limited. However, free tissue transfer can be an excellent option if local tissue transfer with a pedicled or propeller flap is unsuitable. Although free tissue transfer is the traditional option for lower extremity reconstruction, it is tedious and requires complex technical expertise [12]. Muscle flaps have been used for decades due to their rich blood supply and anti-infection capabilities. In addition, muscle tissues are not only suitable for the obliteration of dead space in complex three-dimensional defects, but can expedite bone healing during the early phases of repair. However, the application of muscle flaps gives rise to an unflattering appearance, interferes with daily functions, or secondary debulking procedures [13–15] leading to prolonged hospital time, additional suffering, and higher cost.

With recent progress in perforator techniques, attention is directed towards improved methods of reconstruction. The peroneal artery perforator flap is a promising option for reconstruction of the lower limbs, especially for coverage of ankle and heel defects [16, 17].

In our series, the necrosis rate of the peroneal artery perforator flap was 18.2% (*N* = 10, including nine men and one woman; mean age, 58.2 years) (Table 2). In the series, seven patients have DM (diabetes mellitus), four patients have HTN (hypertension), and four patients have arteriopathy. Bekara et al. [18] identified the following risk factors: age older than 60 years, diabetes, and arteriopathy, which play a significant role in the rebuilding procedure. In our study, these factors also played an important role in flap necrosis. In addition, all ten cases had smoking histories. We believe smoking is an important risk factor that jeopardized the perforators during rotation. Hence, before the procedure, the clinical history should be explored and the smoking status should be documented. Postoperatively, the wound should be monitored periodically. Flap necrosis occurred distally and superficially with small ranges (less than 4 cm^2^); dressing changes may address this issue. If the range is larger, adequate debridement or STSG may be suitable.

**Table 2:** Patients occurred necrosis

Patient No.	Age/gender	Etiology	Smoke history	Size of defect (cm)	Comorbidities	Secondary procedure
6	54/M	Open fracture	Yes	8 × 6	DM/Arteriopathy	Debridement, suture
9	69/F	Osteomyelitis	Yes	12 × 8	DM	STSG
23	66/M	OFSI	Yes	6 × 3	HTN	STSG
26	44/M	Machine crashed	Yes	6 × 6	HTN	STSG
28	44/M	Open fracture	Yes	5 × 15	Arteriopathy	Dressing change
29	60/M	Chronic tophus gout	Yes	3 × 4	Chronic tophus	STSG
31	48/M	OFSI	Yes	10 × 8	DM	STSG
37	78/M	Open fracture	Yes	15 × 15	DM/HTN/arteriopathy	STSG
45	61/M	OFSI	Yes	4 × 6	DM/arteriopathy	Debridement, suture
55	58/M	Crush-injured	Yes	15 × 10	DM/HTN	STSG

Notes: *M* male, *F* female, *OFSI* open fractures with secondary infection, *DM* diabetes mellitus, *HTN* hypertension, *STSG* splint thickness skin graft

According to the authors’ experiences, the ratio of flap length to width, the condition of the pedicle, and the proper thinning of flap may have an important influence on flap survival. As we all know, the length-to-width ratio of random skin flap must not exceed 2:1; otherwise, ischemia and necrosis of the distal flap may occur [19]. However, the length-to-width ratio of perforator flap also existed. In our series, we found that the ratio should not exceed 8:1. When the pattern of the harvested flap exceeded the limit, the distal blood supply would be affected. Besides, the pedicle was of equal importance in perforator flap survival. When the pedicle was identified, soft tissue around it must be wiped off thoroughly under the premise that blood supply would not be affected because during the rotation of the perforator, the redundant tissue might menace the blood transmission leading to descend flap survival rate. Occasionally, two or more perforators appeared at the same time. In our experiences, more perforators were not good for flap survival; on the contrary, they may be harmful, for the reason that one of which was likely to twist or surrounding tissue oppress the pedicle resulting in flap failure in the rotation process. Once flap blood supply was influenced, it was necessary to take out stitches and put the harvested flap back instantly, and a delay transfer procedure was done until the blood supply of the harvested flap improved obviously. Therefore, we usually reserve only one perforator, and it must be the distal one in order to gain adequate blood supply, increase flap survival rate, and enhance the repairable scope. With regard to small defect or relative wide defect (length-to-width ratio less than 4:1), fearless debulking procedure can be done because of the good capillary network, while to big defect or relative long defect (length-to-width ratio more than 4:1), cautious debulking procedure can be done or only margin fat granule can be removed. Moreover, vasodilator was not used in any patient, and we concluded that flap survival may not be related to the application of vasodilator.

However, there were several limitations in our study, particularly the inadequate data collection such as the length of the pedicle, the location of the pedicle, and function and appearance quantized evaluation. In our further research, these limitations will be put in an important position.

In the lower limbs involving the anterior tibial area, ankle, heel area, or the dorsum of the foot, thin, pliable, durable, and gliding soft tissue transfer is the preferred option to achieve a satisfactory esthetic outcome. The peroneal artery perforator flap has these characteristics and is an adequate candidate for this program. In addition, the peroneal artery perforator flap is a time efficient, esthetic, and reliable procedure with lower donor site morbidity, enabling significant coverage for chronic infection, and it can sustain ancillary surgical procedures. Previous studies have shown that the settlement of chronic osteomyelitis and infected wounds is dependent on adequate debridement and extermination of dead spaces; in contrast, the type of flap used to reconstruct lower extremity defects has little impact on the ultimate result [20–22]. Aggressive debridement and eradication of dead spaces with an effective flap must be used when treating chronic wounds.

In summary, the peroneal artery perforator flap is a beneficial and reliable technique; it is appropriate for small to moderate extremity defects, especially in the ankle and heel.

## Conclusion

The peroneal artery perforator flap is a reliable and reproducible procedure providing low postoperative morbidity, good daily functions, and relatively satisfactory esthetic results, without sacrificing any major vessels or nerves. It is intended to be a suitable alternative for the reconstruction of lower limb defects. Because microvascular anastomosis is not required, the flap is less time consuming and has a lower risk of vascular thrombosis compared with other complex techniques. Hence, the peroneal artery perforator flap is a reliable option for the treatment of chronic lower extremity wounds.

## Abbreviations

ATRSI: Achilles tendon rupture with secondary infection
DM: Diabetes mellitus
F: Female
HTN: Hypertension
M: Male
OFSI: Open fractures with secondary infection
STSG: Splint thickness skin graft

## References

[1] Checcucci G, Galeano M, Zucchini M, Zampetti PG, Ceruso M (2014). Reverse flow first dorsal metacarpal artery flap for covering the defect of distal thumb. Microsurgery.

[2] Wang X, Mei J, Pan J, Chen H, Zhang W, Tang M (2013). Reconstruction of distal limb defects with the free medial sural artery perforator flap. Plast Reconstr Surg.

[3] Zhu YL, Yi WMM, Xiao-Qing HMM, Min ZMM, Li FB, Xu YQ (2013). Foot and ankle reconstruction: an experience on the use of 14 different flaps in 226 cases. Microsurgery.

[4] Chen C, Tang P, Zhang X (2014). The dorsal homodigital island flap based on the dorsal branch of the digital artery: a review of 166 cases. Plast Reconstr Surg.

[5] Liu J, Zheng H (2014). Free distal ulnar artery perforator flaps for the reconstruction of a volar defect in fingers. J Plast Reconstr Aesthet Surg.

[6] Hallock GG (2014). Medial sural artery perforator free flap: legitimate use as a solution for the ipsilateral distal lower extremity defect. J Reconstr Microsurg.

[7] Yoshimura M, Imura S, Shimamura K, Yamauchi S, Nomura S (1984). Peroneal flap for reconstruction in the extremity: preliminary report. Plast Reconstr Surg.

[8] Taylor GI, Pan WR (1998). Angiosomes of the leg: anatomic study and clinical implications. Plast Reconstr Surg.

[9] Blondeel PN, Van Landuyt KH, Monstrey SJ (2003). The “Gent” consensus on perforator flap terminology: preliminary definitions. Plast Reconstr Surg.

[10] Tanaka K, Matsumura H, Miyaki T, Watanabe K (2006). An anatomic study of the intermuscular septum of the lower leg; branches from the posterior tibial artery and potential for reconstruction of the lower leg and the heel. J Plast Reconstr Aesthet Surg.

[11] Wei FC, Mardini S (2004). Free-style free flaps. Plast Reconstr Surg.

[12] Levin LS (2006). Foot and ankle soft-tissue deficiencies: who needs a flap?. Am J Orthop.

[13] Chowdary RP, Murphy RX (1992). Delayed debulking of free muscle flaps for aesthetic contouring debulking of free muscle flaps. Br J Plast Surg.

[14] AF P, Unal MB, Cansü E (2011). The reconstruction of foot soft tissue defects by tangential debulking of the latissimus dorsi flap. J Reconstr Microsurg.

[15] Parrett BM, Boumerhi JS, Buntic RF, Safa B, Buncke GM, Brooks D (2010). Refining outcomes in dorsal hand coverage: consideration of aesthetics and donor-site morbidity. Plast Reconstr Surg.

[16] TC L, Lin CH, Lin CH, Lin YT, Chen RF, Wei FC (2011). Versatility of the pedicled peroneal artery perforator flaps for soft-tissue coverage of the lower leg and foot defects. J Plast Reconstr Aesthet Surg.

[17] Ribuffo D, Atzeni ML, Guerra M (2010). Clinical study of peroneal artery perforators with computed tomographic angiography: implications for fibular flap harvest. Anat Clin.

[18] Bekara F, Herlin C, Mojallal A (2016). A systematic review and meta-analysis of perforator-pedicled propeller flaps in lower extremity defects. Plast Reconstr Surg.

[19] Cai L, Huang W, Lin D (2014). Effects of traditional Chinese medicine Shuxuetong injection on random skin flap survival in rats. ScientificWorldJournal.

[20] Hong JP, Shin HW, Kim JJ, Wei FC, Chung YK (2005). The use of anterolateral thigh perforator flaps in chronic osteomyelitis of the lower extremity. Plast Reconstr Surg.

[21] Rodriguez ED, Bluebond-Langner R, Copeland C, Grim TN, Singh NK, Scalea T (2009). Functional outcomes of posttraumatic lower limb salvage: a pilot study of anterolateral thigh perforator flaps versus muscle flaps. J Trauma.

[22] Yildirim S, Gideroğlu K, Aköz T (2003). The simple and effective choice for treatment of chronic calcaneal osteomyelitis: neurocutaneous flaps. Plast Reconstr Surg.

